# Author Correction: Pan-cancer circulating tumor DNA detection in over 10,000 Chinese patients

**DOI:** 10.1038/s41467-021-21285-2

**Published:** 2021-02-10

**Authors:** Yongliang Zhang, Yu Yao, Yaping Xu, Lifeng Li, Yan Gong, Kai Zhang, Meng Zhang, Yanfang Guan, Lianpeng Chang, Xuefeng Xia, Lin Li, Shuqin Jia, Qiang Zeng

**Affiliations:** 1 Health Management Institute, The Second Medical Center & National Clinical Research Center for Geriatric Diseases, Chinese PLA General Hospital, Beijing, 100089 P. R. China; 2 Department of Medical Oncology, The First Affiliated Hospital of Xi’an Jiaotong University, Xi’an, 710061 Shanxi P. R. China; 3 Geneplus-Beijing Institute, Beijing, 102206 P. R. China; 4 Department of Cancer Prevention, National Cancer Center/Cancer Hospital, Chinese Academy of Medical Sciences and Peking Union Medical College, Beijing, 100010 P. R. China; 5 Department of Molecular Diagnostics, Key Laboratory of Carcinogenesis and Translational Research (Ministry of Education/Beijing), Peking University Cancer Hospital & Institute, Beijing, 100142 P. R. China; 6 Department of Medical Oncology, Beijing Hospital, National Center of Gerontology, Beijing, 100010 P. R. China; 7 Institute of Geriatric Medicine, Chinese Academy of Medical Sciences, Beijing, 100010 P. R. China

**Keywords:** Cancer genomics, DNA sequencing

Correction to: *Nature Communications* 10.1038/s41467-020-20162-8, published online 4 January 2021.

This Article contained errors in the “Methods” section. In the first line of the “Methods” section, the samples were incorrectly described as “Between January 2017 to July 2019, a total of 12,337 patients with >40 different cancer types were enrolled at Geneplus Medical Laboratory (Beijing, China)”. The correct version reads “Between January 2017 to June 2019, a total of 12,337 patients with >40 different cancer types were enrolled at Geneplus Medical Laboratory (Beijing, China)”.

Additionally, in the “Methods” section entitled “Targeted deep sequencing for circulating cfDNA and WBC DNA” in the second paragraph, second sentence, it was incorrectly stated that “Afterward, all libraries were hybridized to self-built probes, and DNA sequencing was performed using the MGISeq-2000 Sequencing System (BGI, Shenzhen, China) per the manufacturer’s guidelines”. The correct sentence states “Afterward, all libraries were hybridized to self-built probes, and DNA sequencing was performed using the Gene+Seq-2000 sequencing system (GenePlus-Suzhou, Suzhou, China) per the manufacturer’s guidelines”. These errors have been corrected in the PDF and HTML versions of the Article.

