# Supplementary material for: Pan-cancer circulating tumor DNA detection in over 10,000 Chinese patients
Source: Nat Commun. 2021 Jan 4;12:11. doi: 10.1038/s41467-020-20162-8 (PMC7782482; doi:10.1038/s41467-020-20162-8)
Supplement: Supplementary file 1 — Supplementary Information [file 41467_2020_20162_MOESM1_ESM.pdf]

Supplementary Figures

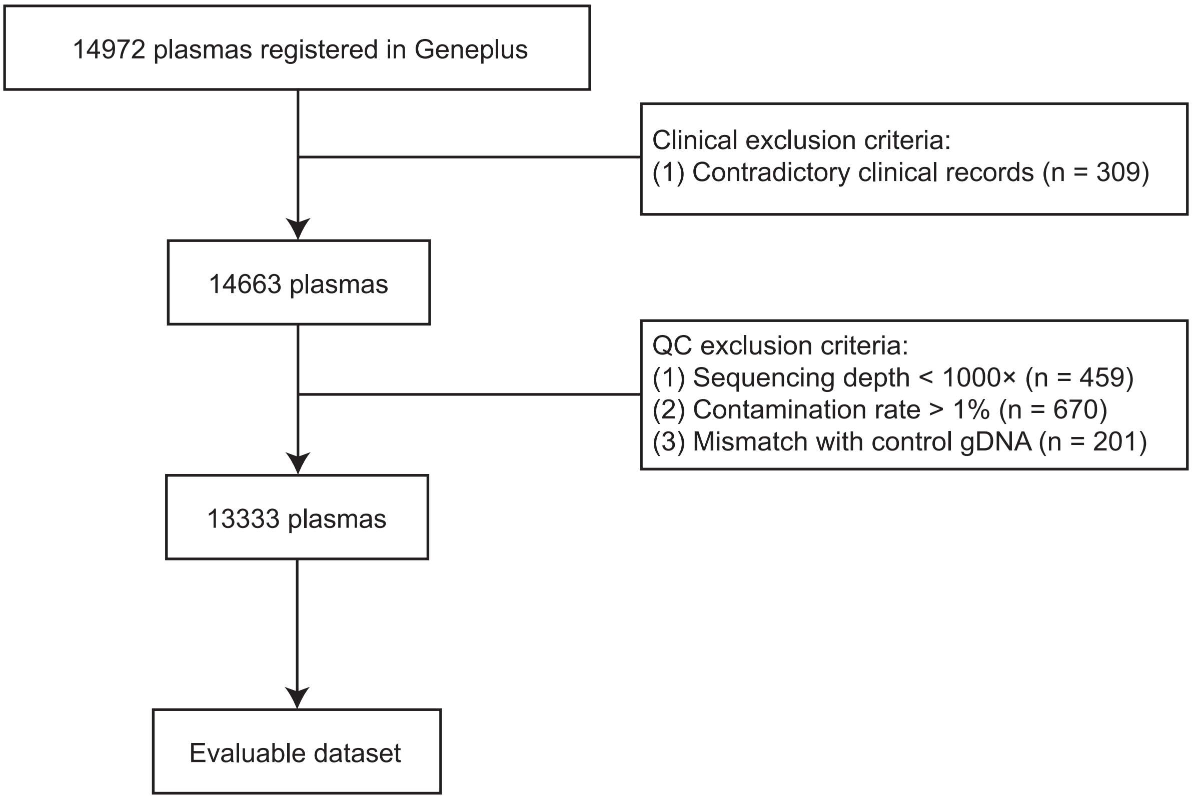

Fig. S1. Sample selection and exclusion criteria.

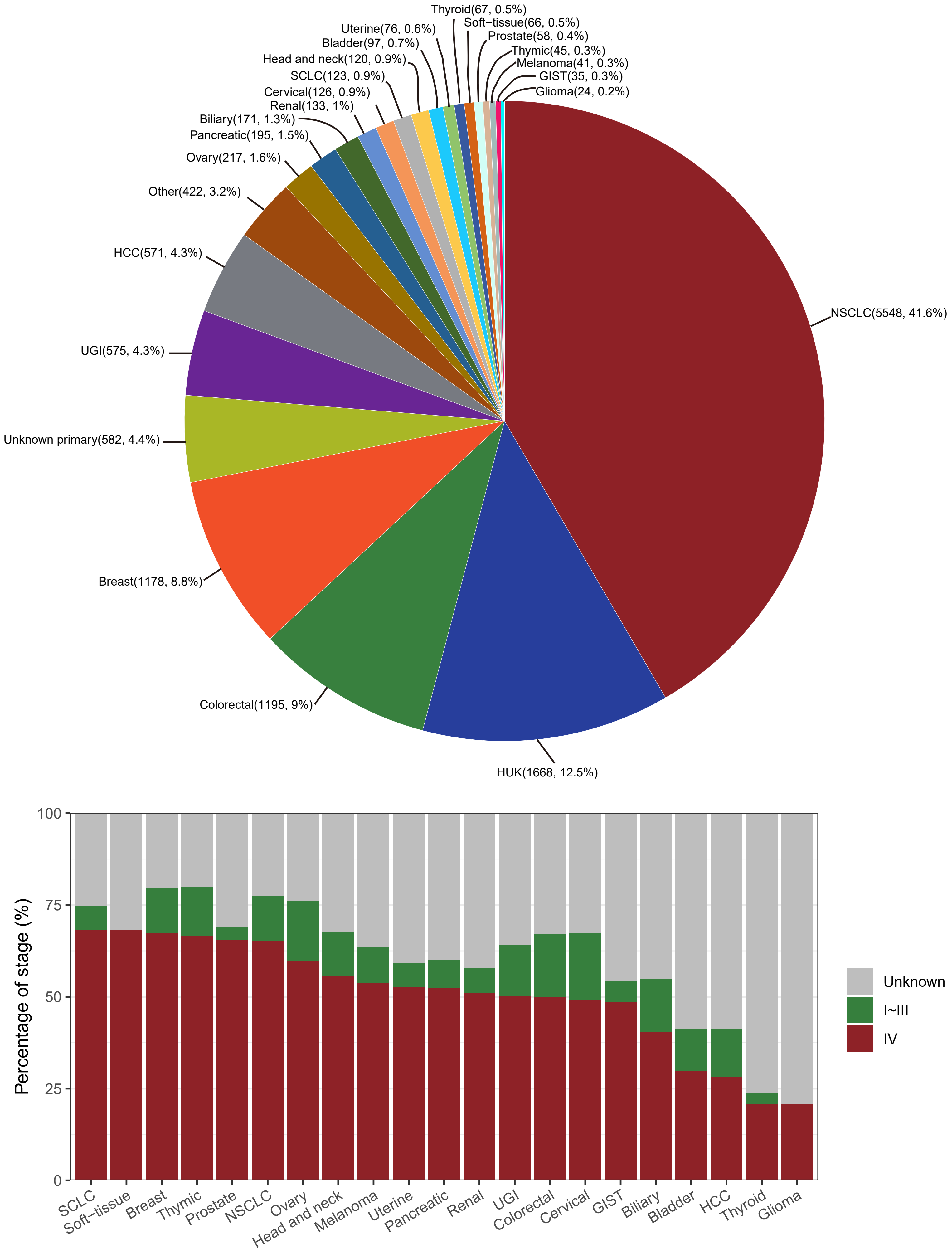

Fig. S2. The distribution of blood sample according to cancer types and clinical stages. GIST, gastrointestinal stromal tumor; HCC, hepatocellular carcinoma; HUK, histology-unknown lung cancer; NSCLC, non-small cell lung cancer; SCLC, small cell lung cancer; UGI, upper gastrointestinal cancer.

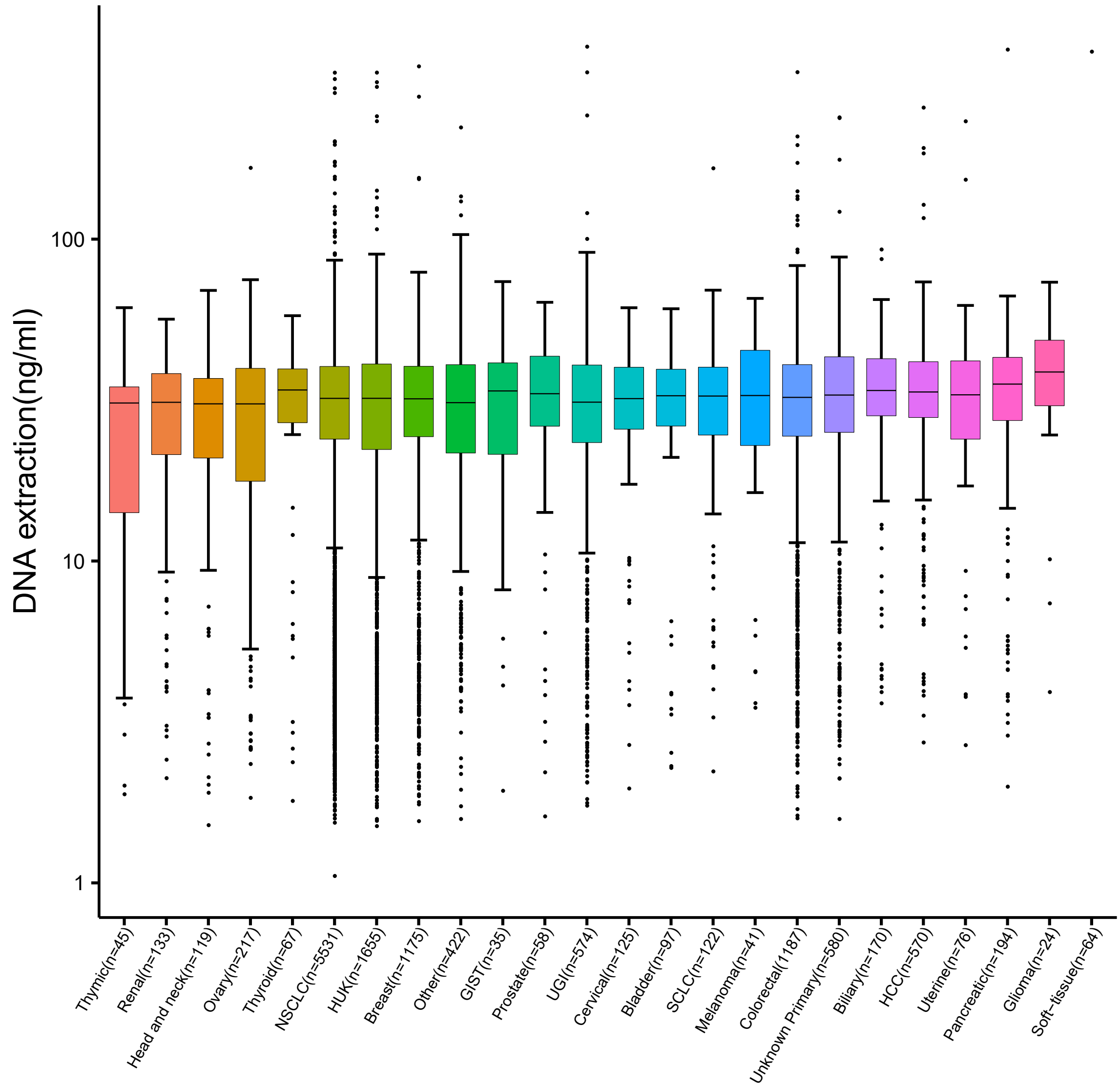

Fig. S3. Blood cfDNA extraction concentration in different cancer types. Centre line, median; box limits, upper and lower quartiles; whiskers, 1.5x interquartile range; points, outliers. GIST, gastrointestinal stromal tumor; HCC, hepatocellular carcinoma; HUK, histology-unknown lung cancer; NSCLC, non-small cell lung cancer; SCLC, small cell lung cancer; UGI, upper gastrointestinal cancer.

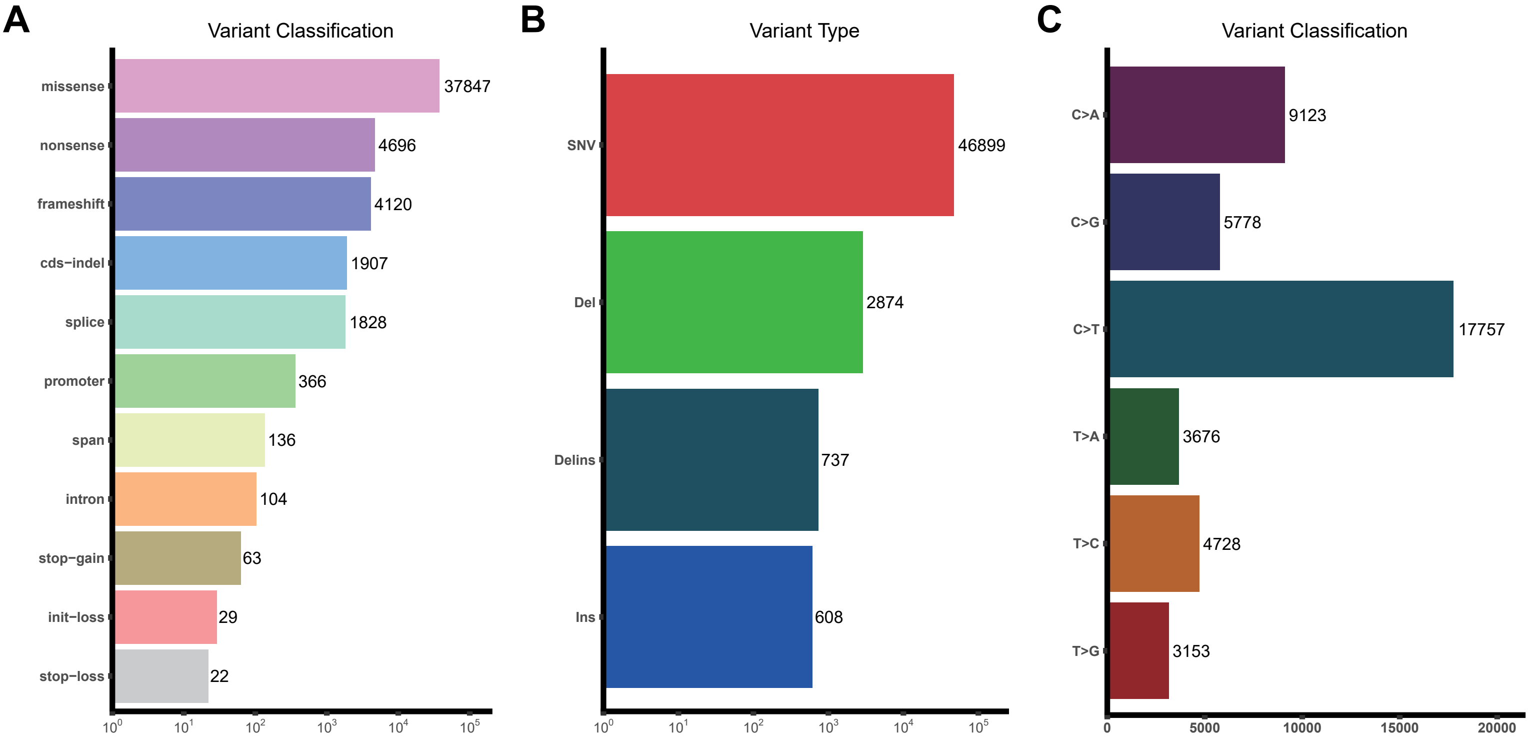

Fig. S4. The classification of all detected SNVs and Indels. SNVs and Indels are classified according to genetic changes and genomic sites (A), the property of base changes (B), and specific base change types for SNVs (C). SNV, single nucleotide variant; Indel, small fragment of insertion or deletion; Del, deletion; Ins, insertion; Delins, fragment deletion coupling with insertion.

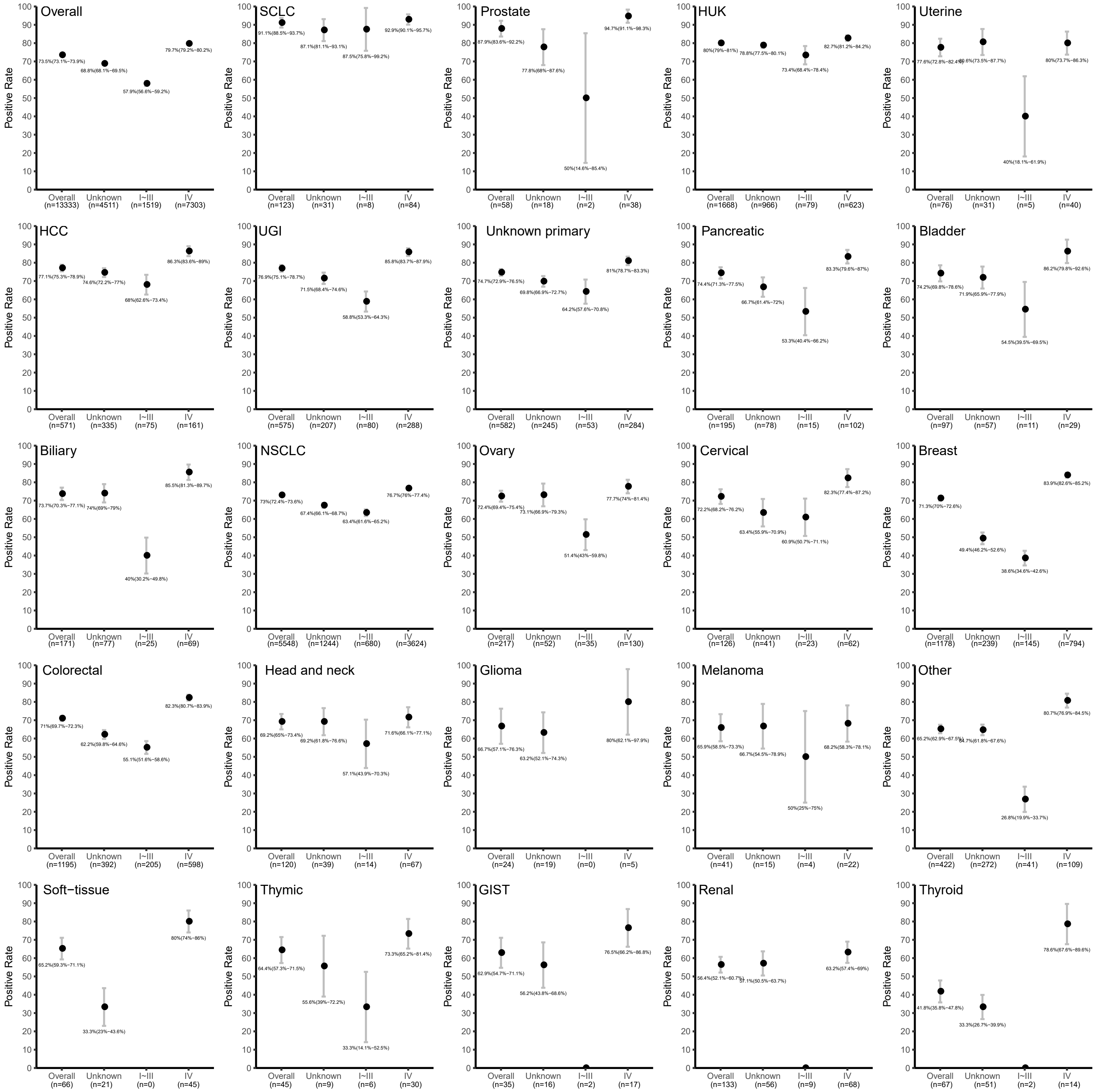

Fig. S5. The detection sensitivity of ctDNA in multiple cancer types with different clinical stages. Error bars indicate the SEM of ctDNA postive rates. GIST, gastrointestinal stromal tumor; HCC, hepatocellular carcinoma; HUK, histology-unknown lung cancer; NSCLC, non-small cell lung cancer; SCLC, small cell lung cancer; UGI, upper gastrointestinal cancer.

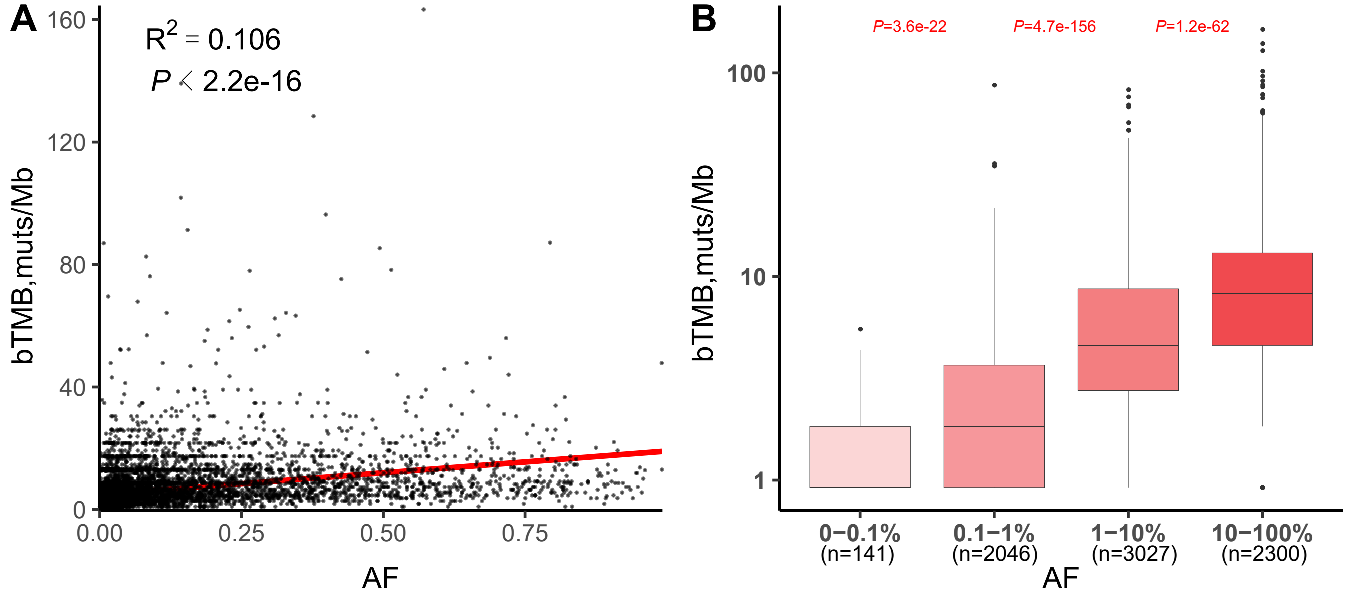

Fig. S6. Correlation between blood tumor mutational burden (bTMB) and allele frequency (AF). (A) ctDNA AFs and bTMB are weakly linear-dependent according to two-tailed Pearson Correlation test. (B) The level of bTMB in different subgroups divided by ctDNA AF, which is listed on the x-axis. Centre line, median; box limits, upper and lower quartiles; whiskers, 1.5x interquartile range; points, outliers. Statistics is performed using two-sided Mann-Whitney U test.

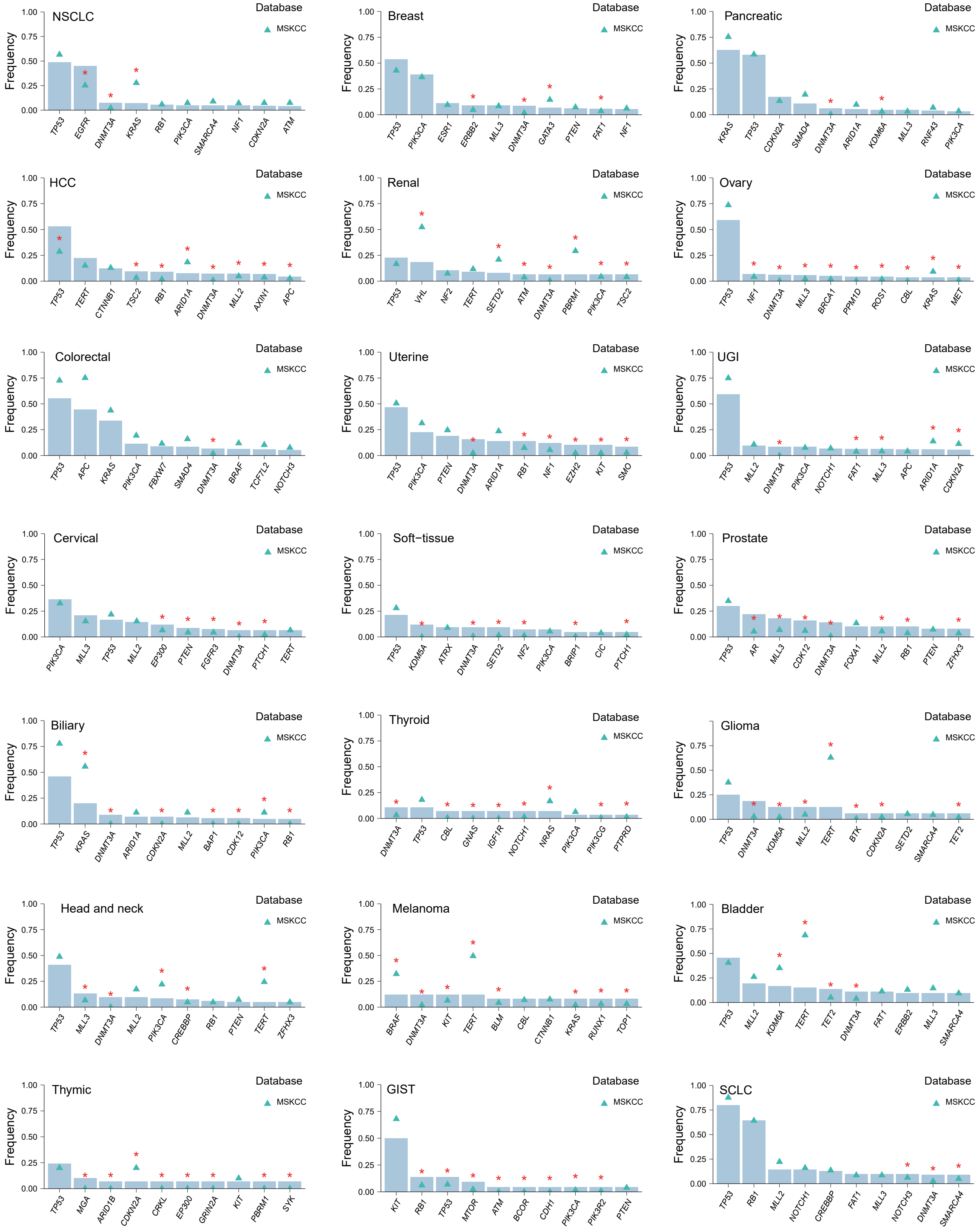

Fig. S7. Prevalence of gene alterations in the ctDNA cohort and MSKCC by cancer types. The columns and green triangles represent the prevalence of mutant genes in the ctDNA cohort and MSKCC, respectively. The genes that are significantly discrepant between both cohorts (two-sided Fisher's exact test) and exhibit > 50%-fold change are labeled as red asterisks.

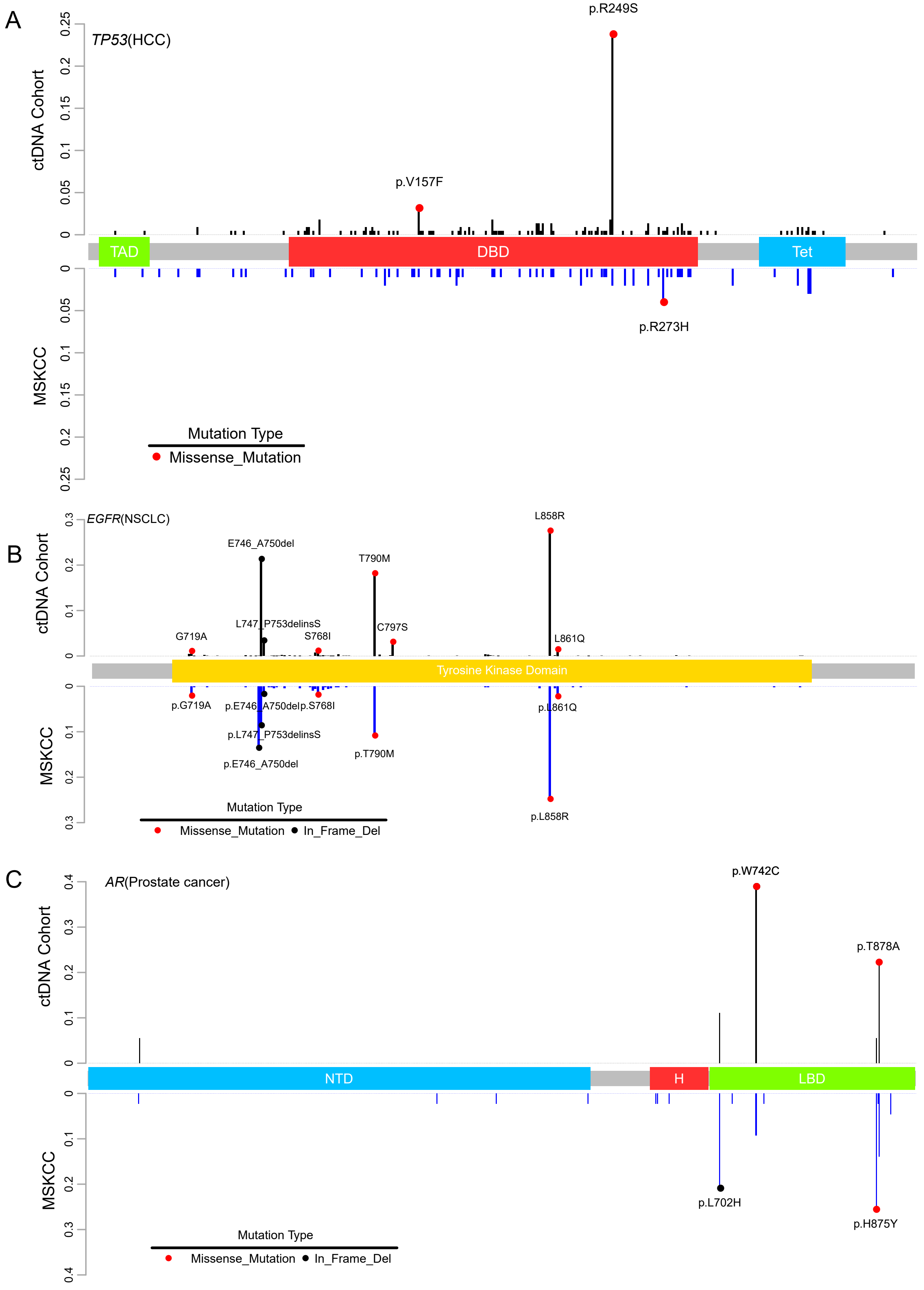

Fig. S8. The distribution of TP53 mutations in HCC (A), EGFR mutations in lung cancer (B), and AR mutations in prostate cancer (C). The upper and lower bars in each panel reflect the relative frequency of different mutational sites in the ctDNA cohort and MSKCC, respectively.

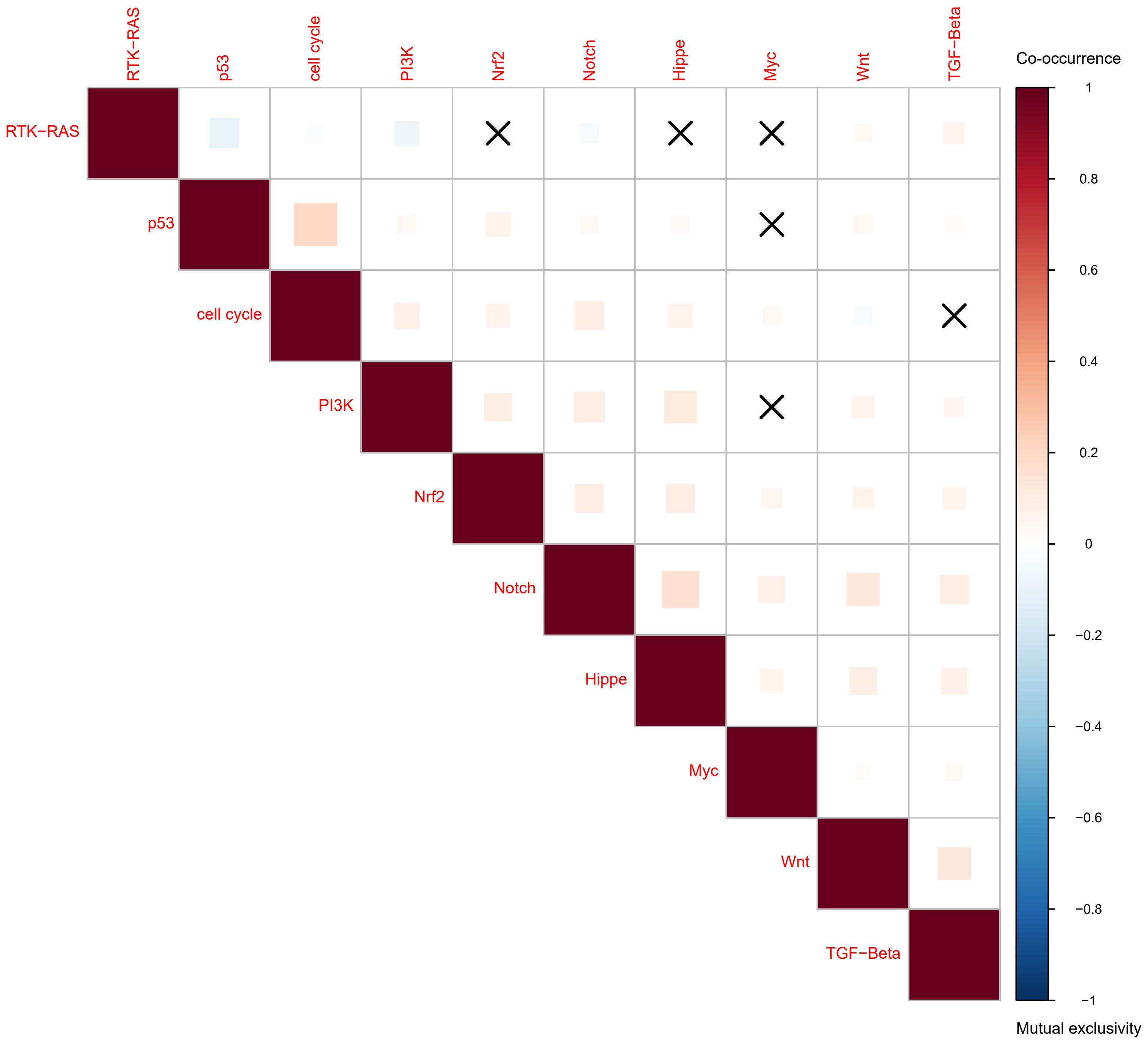

Fig. S9. Mutual exclusivity and co-occurrence among pathway alterations. Mutual exclusivity (blue) and co-occurrence (brown) of gene alterations between different pathways. "×" indicates no significant relationships.

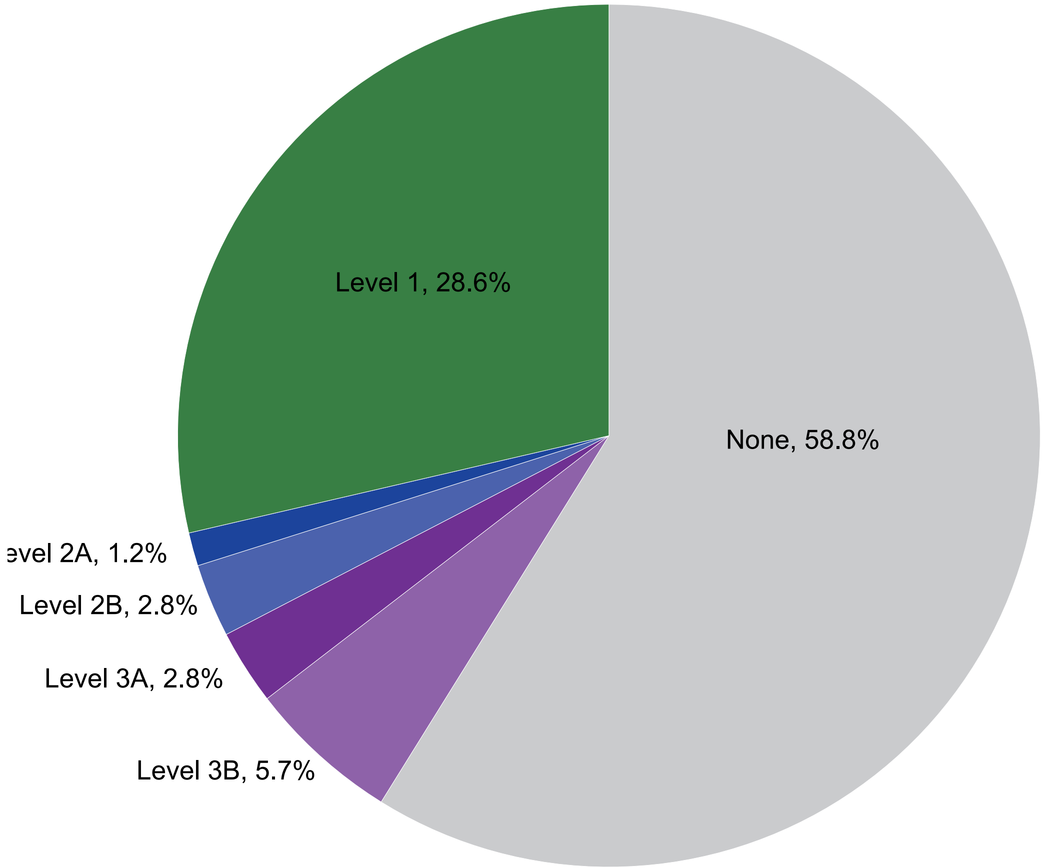

Fig. S10. Prevalence of samples with different level of drug-sensitive mutations. Alterations are annotated based on their clinical actionability according to OncoKB, and samples are assigned by the highest level of actionable alteration.

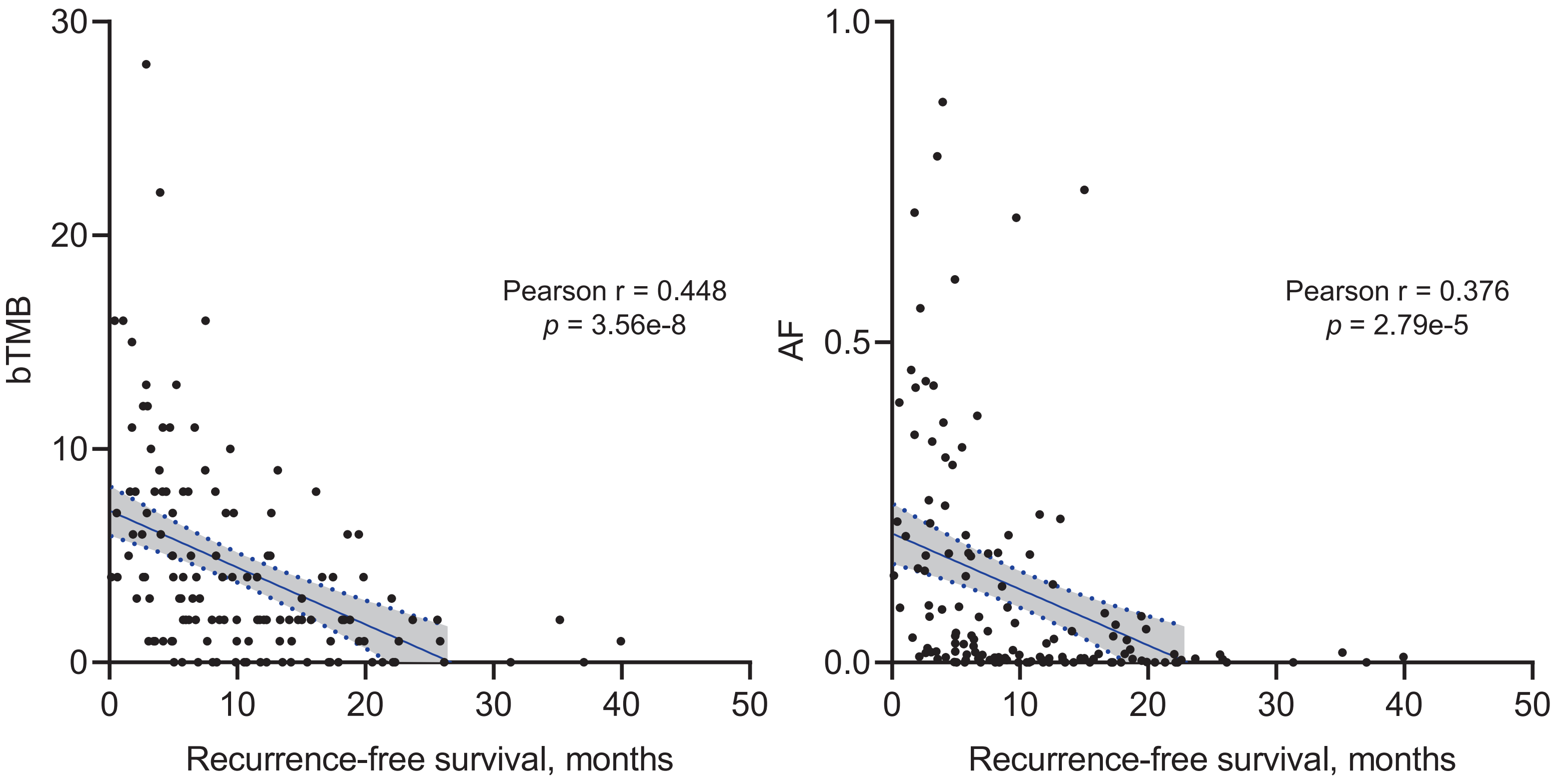

Fig. S11. bTMB and ctDNA AF are negatively correlated with RFS. Pearson Correlation Coefficient is used to evaluate the linear correlation. Error bars indicate the 95% confidence intervals of trend lines.
