# Supplementary material for: Pan-cancer circulating tumor DNA detection in over 10,000 Chinese patients
Source: Nat Commun. 2021 Jan 4;12:11. doi: 10.1038/s41467-020-20162-8 (PMC7782482; doi:10.1038/s41467-020-20162-8)
Supplement: Supplementary file 3 — Description of Additional Supplementary Files [file 41467_2020_20162_MOESM3_ESM.pdf]

Description of Additional Supplementary Files

File Name: Supplementary Data 1
Description: Capture probes design.

File Name: Supplementary Data 2
Description: General clinical information of analytical cohort.

File Name: Supplementary Data 3
Description: CfDNA mutation list, including CH variations.

File Name: Supplementary Data 4
Description: Copy number variation list.

File Name: Supplementary Data 5
Description: Chromosomal rearrangement list.

File Name: Supplementary Data 6
Description: Source determination of cfDNA variants.

File Name: Supplementary Data 7
Description: Actionable alteration list.

File Name: Supplementary Data 8
Description: Samples with available prognosis.
