# Supplementary material for: Pan-cancer circulating tumor DNA detection in over 10,000 Chinese patients
Source: Nat Commun. 2021 Jan 4;12:11. doi: 10.1038/s41467-020-20162-8 (PMC7782482; doi:10.1038/s41467-020-20162-8)
Supplement: Supplementary file 12 — Reporting Summary [file 41467_2020_20162_MOESM12_ESM.pdf]

nature research

Corresponding author(s): Qiang Zeng

Last updated by author(s): Oct 13, 2020

Reporting Summary

Nature Research wishes to improve the reproducibility of the work that we publish. This form provides structure for consistency and transparency in reporting. For further information on Nature Research policies, see Authors & Referees and the Editorial Policy Checklist .

Statistics

For all statistical analyses, confirm that the following items are present in the figure legend, table legend, main text, or Methods section.

n/a	Confirmed	
☐	☒	The exact sample size (*n*) for each experimental group/condition, given as a discrete number and unit of measurement
☐	☒	A statement on whether measurements were taken from distinct samples or whether the same sample was measured repeatedly
☐	☒	The statistical test(s) used AND whether they are one- or two-sided<br>*Only common tests should be described solely by name; describe more complex techniques in the Methods section.*
☒	☐	A description of all covariates tested
☒	☐	A description of any assumptions or corrections, such as tests of normality and adjustment for multiple comparisons
☐	☒	A full description of the statistical parameters including central tendency (e.g. means) or other basic estimates (e.g. regression coefficient) AND variation (e.g. standard deviation) or associated estimates of uncertainty (e.g. confidence intervals)
☐	☒	For null hypothesis testing, the test statistic (e.g. *F*, *t*, *r*) with confidence intervals, effect sizes, degrees of freedom and *P* value noted<br>*Give P values as exact values whenever suitable.*
☒	☐	For Bayesian analysis, information on the choice of priors and Markov chain Monte Carlo settings
☒	☐	For hierarchical and complex designs, identification of the appropriate level for tests and full reporting of outcomes
☐	☒	Estimates of effect sizes (e.g. Cohen's *d*, Pearson's *r*), indicating how they were calculated

Our web collection on statistics for biologists contains articles on many of the points above.

Software and code

Policy information about availability of computer code

Data collection	Burrows-Wheeler Aligner (v0.7.12-r1039) tool was used to align clean reads to the reference human genome with default parameters. Duplicate reads were identified and marked with Picard's Mark Duplicates tool (v2.6.0). The Gene Analysis Toolkit (v3.6-0-g89b7209) was used to perform local realignment and base quality recalibration. Somatic single-nucleotide variations and insertions or deletions of small fragments were called using the MuTect2 (v3.4–46-gbc02625). Copy number was estimated by Contra algorithm (v2.0.8).
Data analysis	The consistency between two continuous variables was assessed using Pearson correlation analysis. The proportional compositions of two or more variables were compared using Chi-square or Fisher's exact tests. Mann-Whitney U tests were used for the comparison of means between two datasets. Kaplan-Meier survival analysis was used to evaluate the association between bTMB/ctDNA AF and PFS. All statistical analyses were performed using SPSS 22.0 (IBM, Armonk, NY, USA). All tests were two-sided, and p-values <0.05 were considered statistically significant.

For manuscripts utilizing custom algorithms or software that are central to the research but not yet described in published literature, software must be made available to editors/reviewers. We strongly encourage code deposition in a community repository (e.g. GitHub). See the Nature Research guidelines for submitting code & software for further information.

Data

Policy information about availability of data

All manuscripts must include a data availability statement. This statement should provide the following information, where applicable:

- Accession codes, unique identifiers, or web links for publicly available datasets
- A list of figures that have associated raw data
- A description of any restrictions on data availability

Public databases used in the study include Catalogue of Somatic Mutations in Cancer (COSMIC, http://cancer.sanger.ac.uk/cosmic), The Cancer Genome Atlas (TCGA, https://cancergenome.nih.gov/), dbsnp (https://www.ncbi.nlm.nih.gov/projects/SNP/), 1000G (https://www.1000genomes.org/), ESP6500 (https://evs.gs.washington.edu/), ExAC (http://exac.broadinstitute.org/), and OncoKB (https://www.oncokb.org/). Sequencing data from MSKCC can be obtain in https://www.cbioportal.org/study/summary?id=msk_impact_2017. All study-associated data, including sample information and next-generation sequencing data, are

available through the European Variation Archive (https://www.ebi.ac.uk/ena/browser/view/PRJEB37947). Other study-related data is available in the main text and supplementary materials.

Field-specific reporting

Please select the one below that is the best fit for your research. If you are not sure, read the appropriate sections before making your selection.

- [x] Life sciences
- [ ] Behavioural & social sciences
- [ ] Ecological, evolutionary & environmental sciences

For a reference copy of the document with all sections, see nature.com/documents/nr-reporting-summary-flat.pdf

Life sciences study design

All studies must disclose on these points even when the disclosure is negative.

Sample size

No sample size calculation was performed because this is a retrospective and observational study. All samples including 14,972 peripheral blood samples from 12,337 cancer patients that had received sequencing process were enrolled in this study. To our knowledge, this is so far the largest study regarding circulating tumor DNA from pan-cancer Chinese patients and we think current sample size is sufficient to draw robust conclusions.

Data exclusions

Samples from the same patients with contradictory clinical records were waived (n = 309). Another 1,330 samples with inferior sequencing depth (< 1000×, n = 459), abnormal contamination rate (> 1%, n = 670) for cfDNA, or mismatch between cfDNA and gDNA (n = 201) were excluded from the analytical cohort during data quality control. Altogether, a total of 13,333 blood samples from 11,525 individuals were included in subsequent analyses.

Replication

Most of the results drawn in this study were descriptive. We did not design any experimental measure about replication.

Randomization

The main purposes of this study are to illustrate the genomic landscape of pan-cancer ctDNA and provide a rich resource for following cancer studies. All the finding in the study are observational and can serve as a benchmark to assess the utility of ctDNA and future applications. Based on aforementioned reasons, we did not perform any randomization process in this study.

Blinding

The investigators were blinded to group allocation during data collection, and blinded to cancer types during fundamental data analysis.

Reporting for specific materials, systems and methods

We require information from authors about some types of materials, experimental systems and methods used in many studies. Here, indicate whether each material, system or method listed is relevant to your study. If you are not sure if a list item applies to your research, read the appropriate section before selecting a response.

Materials & experimental systems

n/a	Involved in the study	
x		Antibodies
x		Eukaryotic cell lines
x		Palaeontology
x		Animals and other organisms
	x	Human research participants
x		Clinical data

Methods

n/a	Involved in the study	
x		ChIP-seq
x		Flow cytometry
x		MRI-based neuroimaging

Human research participants

Policy information about studies involving human research participants

Population characteristics	This cohort encompassed 41 principal tumor types. The most contributive type was non-small cell lung cancer (NSCLC, n = 5,548). Other high-contributive types included colorectal cancer (n = 1,195), breast cancer (n = 1,178), upper gastrointestinal cancer (UGI, n = 575), and hepatocellular carcinoma (HCC, n = 571). The primary lesion information was absent for 582 samples labeled as "Unknown primary". Besides, 1,668 lung cancer samples were not recorded with specific histology and labeled as histology-unknown lung cancer (HUK). Within this cohort, over half of plasma samples were obtained from patients with metastatic stage (clinical stage IV, 7,303/13,333, 54.8%), and 11.4% (1,519/13,333) of samples were obtained from patients with localized or regional stages (stage I-III). Besides, the detailed staging information was absent for a considerable proportion of patients (4511/13,333, 33.8%). The median age at diagnose was 58 years (ranged from 5 to 95 years). The number of male and female patients was 6,891 (51.7%) and 6,392 (47.9 %), respectively. The gender information was miss for 50 patients (0.4%).
Recruitment	From January 2017 to July 2019, a total of 12,337 patients with over 40 cancer types were enrolled in this study at Geneplus Medical Laboratory (Beijing, China). The inclusion criteria were as follows: age ≥18 years and ≤80 years; Karnofsky Performance Status (KPS) ≥ 70; clinically diagnosed with malignant tumors that were registered in the study. The exclusion criteria were as follows: KPS <70; patients with multiple primary cancers in distinct regions; being poorly informed about ctDNA sequencing; suffering from other severe complications. All patients received commercial genetic testing using next-generation sequencing platform.<br>Most of the patients were in advanced stages with prior treatment histories. The genomic landscape reveled by this cohort may be different from primary tumors because gene mutations can shift along with treatment pressure to guarantee the survival of tumor cells, and thus may bias the comparison with tissue-based public database.
Ethics oversight	This study was approved by the ethical committee at Chinese PLA General Hospital (S2019-137-01).

Note that full information on the approval of the study protocol must also be provided in the manuscript.
